# Multiple-instance-learning-based detection of coeliac disease in histological whole-slide images

**DOI:** 10.1016/j.jpi.2022.100151

**Published:** 2022-10-28

**Authors:** J. Denholm, B.A. Schreiber, S.C. Evans, O.M. Crook, A. Sharma, J.L. Watson, H. Bancroft, G. Langman, J.D. Gilbey, C.-B. Schönlieb, M.J. Arends, E.J. Soilleux

**Affiliations:** a Lyzeum Ltd, Salisbury House, Station Road, Cambridge CB1 2LA, Cambridgeshire, UK; b Department of Applied Maths and Theoretical Physics, University of Cambridge, Centre for Mathematical Sciences, Wilberforce Road, Cambridge CB3 0WA, Cambridgeshire, UK; c Department of Pathology, University of Cambridge, Tennis Court Road, Cambridge CB2 1QP, Cambridgeshire, UK; d The Alan Turing Institute, 96 Euston Rd, London NW1 2DB, UK; e Oxford Medical School, University of Oxford, S Parks Road, Oxford OX1 3PL, Oxfordshire, UK; f Department of Cellular Pathology, Birmingham Heartlands Hospital, University Hospitals Birmingham, 45 Bordesley Green East, Birmingham B9 5SS, West Midlands, UK; g Division of Pathology, University of Edinburgh, Cancer Research UK Edinburgh Centre, Western General Hospital, Crewe Road South, Edinburgh, EH4 2XR, Lothian, Scotland

**Keywords:** Computational pathology, Deep learning, Weakly supervised learning, Computer vision, Coeliac disease

## Abstract

We present a multiple-instance-learning-based scheme for detecting coeliac disease, an autoimmune disorder affecting the intestine, in histological whole-slide images (WSIs) of duodenal biopsies. We train our model to detect 2 distinct classes, normal tissue and coeliac disease, on the patch-level, and in turn leverage slide-level classifications. Using 5-fold cross-validation in a training set of 1841 (1163 normal; 680 coeliac disease) WSIs, our model classifies slides as normal with accuracy (96.7±0.6)%, precision (98.0±1.7)%, and recall (96.8±2.5)%, and as coeliac disease with accuracy (96.7±0.5)%, precision (94.9±3.7)%, and recall (96.5±2.9)% where the error bars are the cross-validation standard deviation.

We apply our model to 2 test sets: one containing 191 WSIs (126 normal; 65 coeliac) from the same sources as the training data, and another from a completely independent source, containing 34 WSIs (17 normal; 17 coeliac), obtained with a scanner model not represented in the training data. Using the *same-source* test data, our model classifies slides as normal with accuracy 96.5%, precision 98.4% and recall 96.1%, and positive for coeliac disease with accuracy 96.5%, precision 93.5%, and recall 97.3%. Using the *different-source* test data the model classifies slides as normal with accuracy 94.1% (32/34), precision 89.5%, and recall 100%, and as positive for coeliac disease with accuracy 94.1%, precision 100%, and recall 88.2%. We discuss generalising our approach to screen for a range of pathologies.

## Highlights

• Automated detection of coeliac disease.
• Localisation of disease features in histological images.
• WSI classification.
• Inter-scanner/source generalisation.

## Graphical Abstract

**Figure:**
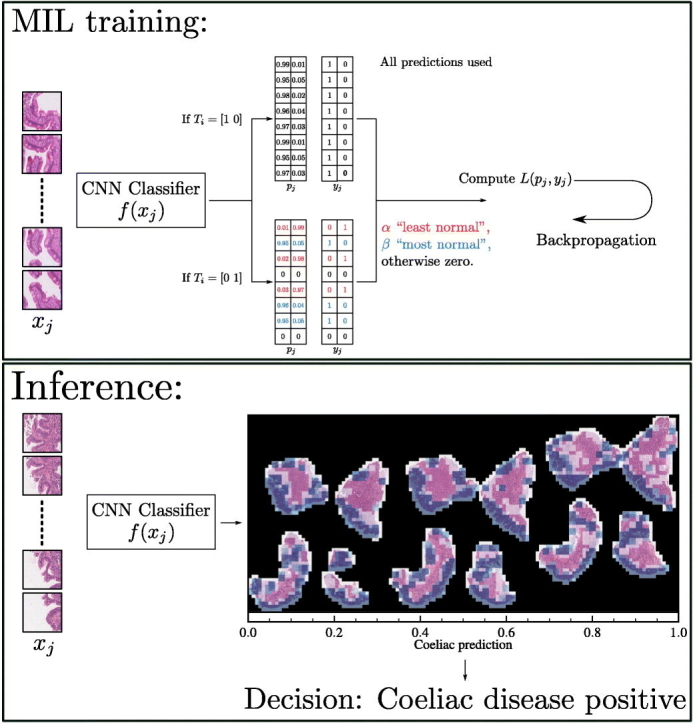

## 1 Introduction

The rise of digital pathology coupled with recent decades of remarkable progress in computer vision^1^^,^^2^ presents exciting new opportunities for the automated detection of diseases and the creation of decision-support tools. Such tools offer the potential to aid pathologists in reporting slides and mitigate the pitfalls associated with manual diagnoses.^2^ Furthermore, realisations that advancing approaches can uncover salient diagnostic features, which are difficult for humans to detect,^3^, ^4^, ^5^ render such tools appealing. Moreover, stark observations concerning pathologist shortages^6^, ^7^, ^8^, ^9^ impress the clear and unmet need for novel tools which can ease the demand on pathologists.

In the histopathological domain, research in this area has largely focused on the detection of cancer (for examples, see Refs^10^, ^11^, ^12^, ^13^, ^14^, ^15^, ^16^, ^17^, ^18^, ^19^, ^20^), which, in many cases, is clearly evident in histological images. However, for some diseases, the histological presentation is less apparent and obtaining a clear diagnosis is more challenging: non-specific disease features can overlap with those of other conditions, may not be spatially localised on slides, and therefore require a pathologist to subjectively weigh a collection of subtle features before issuing a diagnosis. One pathology which fits this description is coeliac disease (CD), which is often diagnosed by inspecting biopsies from the duodenum.

### 1.1 Coeliac disease

Coeliac disease is an autoimmune enteropathy which manifests itself upon the ingestion of gluten—proteins found in wheat, barley, and rye.^21^, ^22^, ^23^, ^24^ Early studies of coeliac disease (CD) first uncovered the connection with food intake by prescribing various dietary restrictions,^23^^,^^25^ before W.-K. Dicke famously reported the success of a wheat-free regime.^26^ The direct link with the gluten component of wheat was made in 1952 by Anderson *et al.*^27^

Approximately, 1.4% of the global population have CD, however the prevalence varies considerably and is difficult to determine precisely.^28^^,^^29^ Interestingly, CD appears to be especially prevalent in certain countries: in Scotland, CD-related hospital admissions are reportedly twice as high as those in England,^30^, ^31^, ^32^ and there is a notable 40-fold difference in the prevalence between Denmark and Sweden.^33^^,^^34^

The symptoms of CD include digestive discomfort, bloating, weight loss, stomach pain, dermatitis herpetiformis (skin rash), fatigue, anaemia, and fertility problems.^35^ In young children, the symptoms include growth retardation, abdominal distension, muscle wasting, and hypotonia.^35^ CD, and in particular a delayed diagnosis, increases the risk of both duodenal adenocarcinoma and lymphoma.^36^^,^^37^ The symptoms are generally alleviated upon the adoption of a gluten-free diet; there is no known cure.^38^

Despite our mature understanding of CD, the procedure for diagnosing it in adult cases is largely underpinned by a pathologist’s manual and subjective interpretation of a duodenal biopsy. For example, the NICE guidelines for England and Wales recommend patients with symptoms suggestive of CD undergo serological testing, which should be followed by a biopsy if either the serology is positive, or if the serology is negative and the symptoms persist.^39^ A comprehensive review from the British Society of Gastroenterology also concludes that biopsies remain essential for adult diagnoses.^40^

Other recent work has argued that, in certain cases, serological information is sufficient to detect CD in adult populations.^41^^,^^42^ Perhaps in the future, more sophisticated approaches will couple such data with pathologist- or computer-vision-based histological analyses to mitigate the high demand on pathologists.

While serological tests are clearly useful as screening tools,^43^, ^44^, ^45^ for the time being, histology-based diagnoses remain the gold-standard.

### 1.2 Histology-based diagnoses

**Fig. 1:**
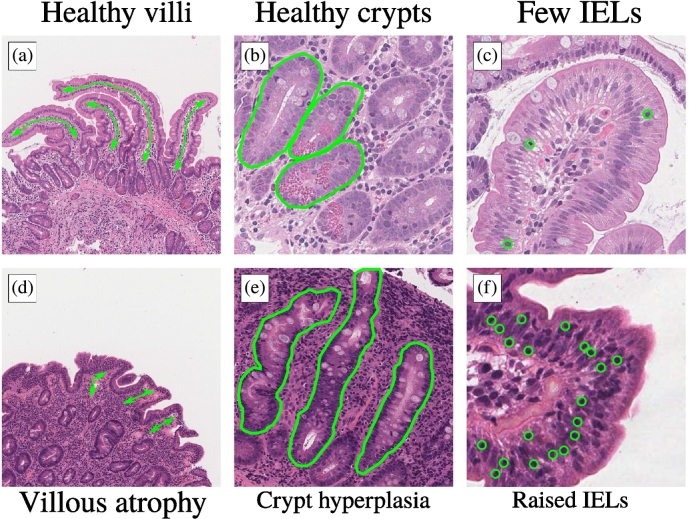
Key differences (demarcated in green) in crypts, villi, and intra-epithelial lymphocytes (IELs) in (a)–(c) normal duodenal tissue and (d)–(f) a positive instance of coeliac disease. Note, in (f) only some, and not all, of the IELs are demarcated. The images are not on uniform spatial scales, and are illustrative only.

In cases of CD, when gluten enters the duodenum, the first part of the digestive tract after the stomach, an autoimmune response effects inflammatory and structural changes which can be used to diagnose the disease (see Fig. 1). These features (see Fig. 1) are mainly atrophy of the villi, hyperplasia of the crypts, and an increase in the number of intra-epithelial lymphocytes. Some studies suggest a ratio of intra-epithelial lymphocytes to intra-epithelial enterocytes of 0.25 or more as evidence of CD.^46^^,^^47^

These features are often assessed using the Marsh–Oberhuber classification scheme,^48^^,^^49^ which provides a scale for assessing the nature and severity of the changes effected by the autoimmune response to gluten (simpler alternatives to the Marsh–Oberhuber scheme also exist^46^^,^^47^).

The challenging and subjective nature of histology-based diagnoses is reflected by the wide variation in quantitative estimates of the inter-observer agreement in CD diagnoses reported in published studies: generally the level of agreement is measured using Cohen’s kappa coefficient,^50^ and is reported to depend on both the biopsy classification scheme used^46^^,^^51^^,^^52^ and the practise setting (i.e., specialist academic analysis, routine diagnostic reporting, and commercial laboratories) in which cases are diagnosed.^53^^,^^54^ Kappa coefficients reported in CD inter-observer agreement studies, which typically involve the interpretation of single samples from D2, vary widely on 0 ≤ *κ* ≤ 1 (see citations for study-specific details).^46^, ^51^, ^52^, ^53^, ^54^, ^55^, ^56^, ^57^, ^58^, ^59^, ^60^, ^61^, ^62^

The poor concordance between observers is understandable when one considers the challenging nature of duodenal biopsies, which contain a panoply of information, including diagnostically important structures that differ in size by orders of magnitude. Moreover, there is a lack of regularity in the preparation of specimens: the samples are prone to damage; the orientation is random; tissue occasionally fails to adhere to the slide; inconsistencies in the staining process give rise to large colour variations across different laboratories—a problem exacerbated by samples cut with non-uniform thickness and (in the case of WSIs) digitisation artefacts imposed by commercial scanners. Furthermore, the observer is restricted to examining two-dimensional slices of inherently three-dimensional structures.

Another substantive hurdle is the variable extent to which evidence of CD is present in the duodenum. While there are clear-cut cases of villous atrophy and crypt hyperplasia—which strongly suggest CD—there are many cases which are subtle: the distinction between coeliac and normal biopsies is not always clear or sharp. Additionally, diagnosing CD from a duodenal biopsy relies on the patient maintaining a gluten-containing diet (otherwise the characteristic changes can alleviate) while suffering from related symptoms—a requirement which is not always met. The NICE guidelines specifically state patients should be advised that insufficient gluten consumption risks a false-negative duodenal biopsy diagnosis.^39^

### 1.3 AI progress

Studies examining the automated detection of CD in a histological setting are relatively scarce. Wei *et al.* present a scheme which involves dividing WSIs into patches of 224×224 pixels (at 20× magnification), labelling the patches based on the patient’s diagnosis and training a convolutional neural network (CNN) to make patch-level classifications.^63^ Wei *et al.* report their model identifies CD, normal tissue, and non-specific duodenitis with accuracies of 95.3%, 91.0%, and 89.2%, respectively.

Despite this good performance, labelling all patches with the slide-level diagnosis has a conceptual limitation: not every patch from a disease-positive biopsy contains evidence of disease, yet every patch is so labelled. Thus, patches containing no evidence of disease, drawn from disease-containing slides, are inadvertently labelled as disease-positive,

Another interesting approach is that of Sali *et al.*, who attempt to determine, using patches, the Marsh–Oberhuber classification of the patient they originate from.^64^ While this work is interesting and displays promising results, it only includes 162 slides from 34 patients, and omits class II (including only classes I, IIIa, IIIb, and IIIc) from the Marsh–Oberhuber scale.

Kowsari *et al.* detail an approach using colour balancing and CNNs to detect coeliac disease in a dataset of 3118 WSIs, taken from a small set of 121 biopsies from 102 patients.^65^ On a test set of 1000 images (from the same underlying dataset), Kowsari *et al.* classify normal slides, coeliac disease, and environmental enteropathy^66^ with ROC AUCs of 0.90, 0.96, and 0.89, respectively.

In this contribution, we apply a weakly supervised multiple instance learning (MIL) method to the problem of diagnosing CD solely from WSIs of duodenal biopsies. Such techniques have shown considerable promise in the detection of tumours in similar contexts^15^^,^^67^ and lend themselves well to situations in which the data have been annotated sparingly: i.e., the realistic setting where only slide- and not pixel-level annotations are available. The overarching idea of our approach is to first predict which regions of a WSI contain evidence of CD disease and to then use these patch-level predictions to map to slide-level classifications.

## 2 Materials and methods

### 2.1 Materials

Our dataset contains of 2075 WSIs from a total of 1175 cases, and includes normal duodenal biopsies as well as biopsies determined positive for coeliac disease (see Table 1). All of the samples are stained with haematoxylin and eosin and come from adults.

**Table 1:** Summary of WSIs from Heartland’s Hospital (H), Addenbrooke’s Hospital (A), and Sheba Medical Centre (S): scans formatted as “.tif” and “.bif” were scanned with a Roche Ventana iScan HT, “.ndpi” a Hamamatsu Nanozoomer XR, “.svs” (the same) Leica Aperio AT2 and “.isyntax” with a Philips IntelliSite Ultra Fast Scanner.

Source	Diagnosis	Set	Cases	Scans				
				“tif”	“.bif”	“.ndpi”	“.svs”	“.isyntax”
H	Normal	Test	21	21	0	17	17	0
		Train	181	176	0	170	170	0
	Coeliac	Test	21	21	6	19	19	0
		Train	208	187	51	188	188	0

A	Normal	Test	64	0	0	0	71	0
		Train	577	0	0	0	645	0
	Coeliac	Test	8	0	0	0	9	0
		Train	61	0	0	0	66	0

S	Normal	Test	17	0	0	0	0	17
		Train	0	0	0	0	0	0
	Coeliac	Test	17	0	0	0	0	17
		Train	0	0	0	0	0	0

The scanned samples originate from 3 sources: Birmingham Heartlands Hospital (University Hospitals Birmingham NHS Foundation Trust, England, UK), Addenbrooke’s Hospital (Cambridge University Hospitals NHS Foundation Trust, England, UK), and Sheba Medical Centre (Tel HaShomer, Israel).

The diagnoses were made by gastrointestinal pathologists practising at the respective hospitals (see Table 1). The cases from Heartlands Hospital were diagnosed as normal if they had no features of CD, increased intra-epithelial lymphocytes, coeliac autoantibodies, malabsorption, diarrhoea, and no gluten-free diet. The cases from Heartlands Hospital diagnosed as CD-positive had clear features of Marsh 2 and above, generally with positive TTG and EMA.

The cases from Addenbrooke’s Hospital were selected from a list of “all-comer” duodenal biopsies, taken in 4 different months. We selected all of the cases described as containing no abnormality (normal) and cases described as consistent with ongoing, active CD (CD-positive) in the pathology reports. The slides from Sheba Medical Centre were all described as containing no abnormalities (normal), or as positive for coeliac disease.

In short, cases listed as normal contained only healthy tissue with no evidence of pathology, and cases listed as CD-positive contained sufficient histological evidence to merit such a diagnosis.

All scans (and accompanying fully anonymised patient data) were obtained with full ethical approval (IRAS: 162057; PI: Dr E. Soilleux).

Our data from Heartlands Hospital is a special case: these research slides were collected specifically for this work and are not the original diagnostic slides—the samples were cut from known cases. In order to capture a diverse range of scanner-specific digitisation artefacts, these samples were scanned on multiple platforms (specified in Table 1).

In the case of the slides from Addenbrooke’s Hospital, some cases have multiple scans because the number of tissue sections cut required more than 1 slide.

In order to access images in Philips’s proprietary “.isyntax” format, we first convert them to RGB “.ome.tiff” files using the tools available from Glencoe Software, Inc: namely “isyntax2raw” and “raw2ometiff”.

### 2.2 WSI preprocessing

*Patch extraction:* The large scale of WSIs prohibits their treatment as single entities, and necessitates their division into small, manageable, patches. For example, Campanella *et al.* observe that 470 WSIs contain roughly the same number of pixels as the *entire* ImageNet data set, which contains in excess of 1.4×10^7^ images (typically resized to 224×224 pixels).^15^^,^^68^ To generate patches, we use QuPath^69^: we extract patches of 256×256 pixels at 10× magnification, corresponding to approximately 10 μm per pixel. The patches are generated using a sliding window approach with a stride of 128 pixels. We use this overlap at both training and inference time. To include only patches which contain a materially significant amount of tissue, we separate foreground from background using Otsu’s threshold^70^ and discard patches containing more than 75% background.

**Fig. 2:**
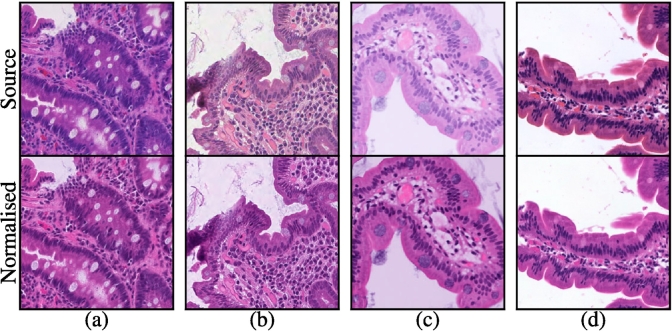
(Top) 256×256 pixel patches at 10× magnification from (a) a Hamamatsu Nanozoomer XR, (b) a Leica Aperio AT2, (c) a Philips IntelliSite Ultra Fast Scanner, and (d) a Roche Ventana iScan HT and (bottom) their normalised equivalents.

*Stain normalisation:* To mitigate the well-known problem of poor generalisation in computational pathology, we standardise the apparent staining characteristics of inputs to our model using Macenko’s method^71^ for stain normalisation. We illustrate the stain normalisation preprocessing step with four examples in Fig. 2.

### 2.3 Multiple instance learning method

Multiple instance learning is becoming increasingly popular in computational pathology (e.g., see Refs^15^^,^^67^). The rationale for choosing a MIL-based approach is that, in the absence of pixel-level annotations, MIL still offers scope for the localisation of predictions (on the patch-level) while only requiring slide-level annotations.^15^^,^^67^

**Fig. 3:**
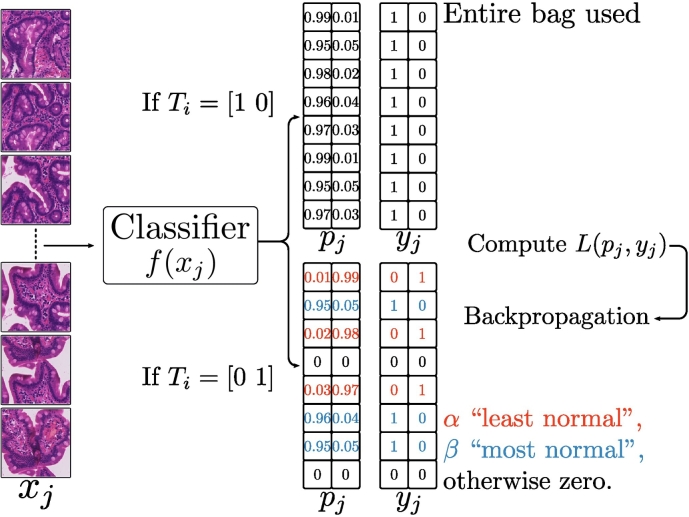
One training step in a multiple instance learning approach: a bag of patches is randomly sampled from a slide; the predictions for each patch are inferred by the classifier; if the slide’s label is positive, the *α* “least normal” patches are labelled positive, the *β* “most normal” patches are labelled negative, and all other predictions and labels are zeroed; if the slide is negative, all patches are labelled negative; backpropagation proceeds in the normal way.

The basic idea involves recognising that, during training, one may safely label all patches from normal slides as normal, but, in the case of disease-positive slides, it is unclear which patches should be labelled as positive and which should be labelled as negative. To address this, one employs a weakly supervised labelling step which associates disease-positive labels with the “least normal” patches sampled from diseased slides. We give a graphical overview of 1 MIL training step in Fig. 3.

In a single training epoch, we visit each slide in random order. Upon visiting a slide, we randomly sample a bag of patches (with replacement) and infer on each patch. If the slide’s label is negative (i.e., normal), we assign a negative proxy label to each patch (see Fig. 3). If the slide’s label is positive (i.e., coeliac disease), we assign positive proxy labels to the *α* “least normal” patches (see Fig. 3), negative proxy labels to the *β* “most normal” patches (see Fig. 3), and zero all other inferences and labels (thus eliminating any gradient flow from these items). Backpropagation then proceeds in the normal way. Lerousseau et al.^67^ gives an excellent description of multiple instance learning in the context of histological images. For all of the results presented in this work, we set *α*=10, *β*=0 and use a bag size of 100 patches.

### 2.4 Model and training details

We implement our model in PyTorch: we choose a ResNet50 classification architecture^72^ initialised with PyTorch’s ImageNet pre-trained weights (with a randomly initialised classification layer), and train for a period 10 epochs using an Adam optimiser with both the learning rate and weight decay set to 10^−4^, and the other parameters set to their defaults.^73^ We use the binary-cross-entropy loss criterion and apply the sigmoid activation function to the classifier’s output.

### 2.5 Metrics

We assess our model’s performance using three metrics: accuracy (the frequency with which the model’s predictions match the slides’ labels), precision (the number of true-positive predictions divided by the total number of positive predictions), and recall/sensitivity/true-positive-rate (the number of true-positive predictions divided by the total number of positive instances of a given class).

Precision and recall are particularly useful when there is a large class imbalance as, unlike accuracy, they account for the number of instances of each class. We present all metrics on the WSI level.

## 3 Results

Our results are organised as follows: in Section 3.1, we detail the development of our model using cross-validation on the training data specified in Table 1; in Section 3.2, we show an example of patch-level predictions overlaid on their corresponding WSI, thus highlighting the regions our model finds indicative of coeliac disease; in Section 3.3, we apply our model to 2 test sets—one containing unseen data from the same sources as the training data (Addenbrooke’s and Heartlands data in Table 1), and another from a truly independent source (Sheba Medical Centre data in Table 1), where the data originate from a centre (using a scanner model/manufacturer) *not* represented in the training data.

### 3.1 Model development

We develop our model using 5-fold cross-validation with the 1841 training WSIs (1161 normal; 680 CD-positive) specified in Table 1. When splitting the data, we (approximately) preserve the training set’s ratio of normal to CD-positive cases in each fold, and impose the condition that scans originating from the same case (patient sample) must always be in the same fold. We treat normal tissue and coeliac disease as 2 distinct classes.

**Fig. 4:**
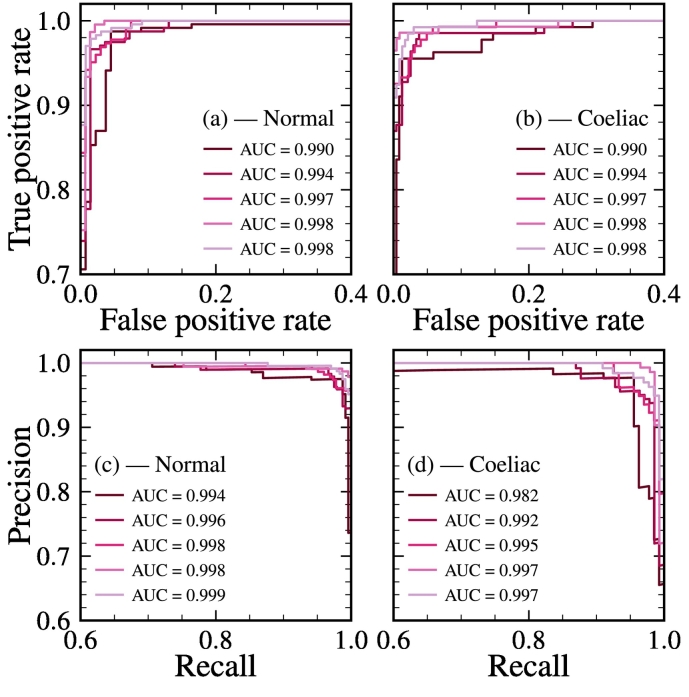
(a)–(b) Receiver operating characteristic and (c)–(d) precision-recall curves for the classification of WSIs as normal and coeliac for each cross-validation fold, respectively.

To summarise the patch-level predictions for a single WSI, we compute their mean. To determine whether WSIs can be classified using the mean of the patch-level predictions, we perform receiver operating characteristic (ROC) (Fig. 4 (a)–(b)) and precision-recall (PR) (Fig. 4 (c)–(d)) analysis on these quantities for each cross-validation fold. In Fig. 4, we see that for both the normal and CD classes, the area under the ROC and PR curves exceeds 0.98 in all cases, which shows the mean of the patch-level predictions is a robust statistic for classifying WSIs.

To foster as general a model as possible, we choose decision thresholds which minimise the difference between the true- and false-positive rates over each of the cross-validation folds. We classify slides as normal if the mean of the normal component of their patch-wise predictions exceeds 0.905, and as CD-positive if the mean of the coeliac component of their patch-wise predictions exceeds 0.096.

We give an overview of our model’s performance on each cross-validation fold, using these thresholds, in Table 2.

**Table 2:** Cross-validation performance of WSI classification.

Class	Fold	Accuracy	ROC AUC	Precision	Recall
Normal	1	0.973	0.990	0.975	0.983
	2	0.971	0.994	0.983	0.971
	3	0.958	0.997	0.995	0.938
	4	0.970	0.998	0.954	1.000
	5	0.965	0.998	1.000	0.949
	**Mean**	0.967	0.995	0.980	0.970

Coeliac	1	0.973	0.990	0.970	0.955
	2	0.968	0.994	0.950	0.964
	3	0.961	0.997	0.911	0.993
	4	0.970	0.998	1.000	0.923
	5	0.965	0.998	0.920	0.992
	**Mean**	0.967	0.995	0.950	0.965

Our cross-validated model classifies slides as normal with accuracy (96.7±06)%, precision (98.0±1.7)%, and recall (96.8±2.5)%, and slides as CD-positive with accuracy (96.7±05)%, precision (94.9±3.7)%, and recall (96.5±2.9)% (the quoted error is the cross-validation standard deviation). It is worth noting here that the cross-validation performance may well be limited by ground-truth noise (see Section 4 for a discussion).

### 3.2 Localisation

To understand what our model finds indicative of coeliac disease, at least on a coarse-grained level, it is informative to overlay the patch-wise predictions on the image as a whole and provide a spatial context to the otherwise disparate patch-wise predictions.

Guided by CD diagnostic criteria,^46^, ^47^, ^48^, ^49^ we expect to find evidence of coeliac disease in patches which contain villi and crypts (see Fig. 1). Evidence of CD should appear on both a structural level, in the form of atrophy of the villi and hyperplasia of the crypts, and on a cellular level, where the prevalence of lymphocytes in the epithelium should be considerably higher than in normal cases.

**Fig. 5:**
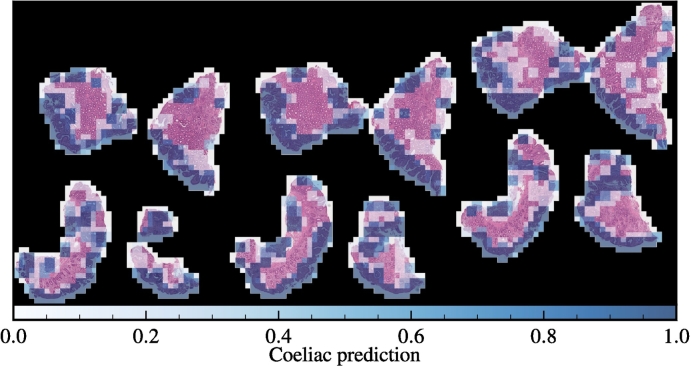
Coeliac component of the patch-level predictions overlayed on the corresponding tissue sections of a slide positive for coeliac disease. The positive predictions are localised to regions containing villous and crypt epithelium. Note: where patches overlap, we display the maximum prediction value in the heatmap; the black regions correspond to discarded “background” patches (see the supplementary material for a larger version of this image, and other examples).

We show an example of such an overlay from a case determined positive for coeliac disease in Fig. 5. The localisation of the predictions in Fig. 5 to regions which pathologists deem diagnostically relevant indicates that our model’s positive patch-level classifications are meaningful, and not spurious. For a larger version of Fig. 5, as well as other normal and CD-positive examples, please see the supplementary material.

### 3.3 Model testing

After developing our model and determining optimal decision thresholds using cross-validation, we train a new instance of the model on all of the training data, again for 10 epochs, and evaluate its performance on 2, small, independent test sets:

1. *Same-source testing—*the scans originate from the same sources as the training data, and were scanned on the same scanners, but were *never* used for the model’s training or development (Heartlands and Addenbrooke’s test data in Table 1).
2. *Different-source testing—*the scans originate from a different source to those used in the model’s training and development (Sheba Medical centre test data in Table 1), and were scanned on a different platform.

In order to clearly distinguish between the performance on test data from the same training source and the test data from a truly independent source, we evaluate the model’s performance on each of these sets separately (see Table 3).

**Table 3:** WSI classification performance for the classes normal (N) and coeliac disease (CD) on independent test data from the same and different sources as the training data.

Source	Class	Accuracy	ROC AUC	Precision	Recall	Scan count
Same	N	0.965	0.996	0.984	0.961	126 N; 74 CD
	CD	0.965	0.996	0.935	0.973	
Different	N	0.941	0.993	0.895	1.000	17 N; 17 CD
	CD	0.941	0.993	1.000	0.882	

The model’s performance on the same-source test set is essentially as good as the cross-validation performance on the training set (96.5% versus 96.7% accuracy), which shows it generalises well to unseen samples from these sources.

In the case of the different-source test set, the model correctly classifies 32 of the 34 cases (94.1%), which suggests it generalises beyond source- and scanner-specific artefacts, has learnt to detect general features of coeliac disease, and makes meaningful classifications.

**Fig. 6:**
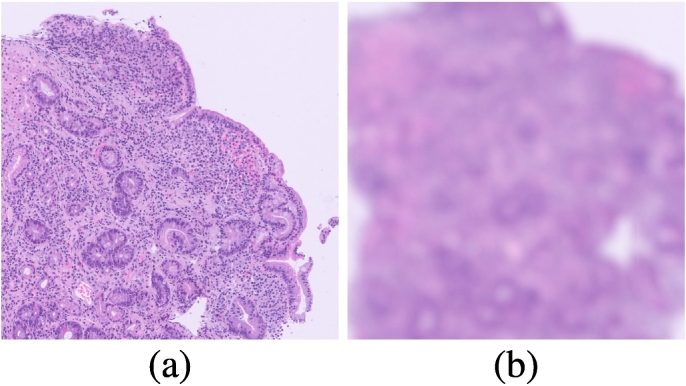
Examples of (a) clearly and (b) poorly focused patches from a test WSI the model failed on (see supplementary material for a downscaled version of this image).

The model only misclassified 2/34 WSIs from the different source test set. In these 2 cases, problems with the scanning procedure have given rise to large regions of blur (out-of-focus image) which obscure 25%–50% of the tissue, thus masking the detail in these areas such that it cannot be resolved. We show an example from a poor quality test WSI in Fig. 6

These 2 misclassifications are an issue of quality control in the data and not a failure to generalise (see Section 4 for discussion).

## 4 Discussion and conclusions

We presented a multiple-instance-learning-based approach for the detection of coeliac disease in histological WSIs of duodenal biopsies. Using cross-validation, our model classified slides as normal with accuracy (96.7±0.6)%, precision (98.0±1.7)%, and recall (96.8±2.5)%, and slides as coeliac with accuracy (96.7±0.5)%, precision (94.9±3.7)%, and recall (96.5±2.9)% (error bars are the cross-validation standard deviation).

Given the large variation typically found in CD inter-observer agreement studies (where pathologists essentially never achieve a complete consensus), it is reasonable to consider that our model’s cross-validation performance is limited by ground-truth noise.

By overlaying the patch-level predictions on a WSI determined positive for coeliac disease, we showed that our model, to a large extent, associates disease-positive predictions with regions containing the structures pathologists consider to be of diagnostic utility in standard practise. The connection between the model’s predictions and structures pathologists consider diagnostically relevant offers evidence that the predictions are meaningful, and not spurious.

We further tested the significance of the model’s predictions, and its ability to generalise, by applying it to 2 unseen test sets: 1 containing data from the same sources as the training data, and 1 from a completely independent source, where the samples originated from a different lab, and were scanned with a scanner model/manufacturer not represented in the training data.

The model’s performance on the same-source test set was essentially as good as the cross-validation performance on the training set (96.5% versus 96.7% accuracy for both classes), which shows the model generalises well to different biopsies from these (same) sources.

In the case of the different source test set, the model correctly classified 32/34 (94%) cases. In the case of the 2 WSIs, the model failed to classify correctly, problems with the scanning procedure gave rise to large regions of blur which substantially obscured significant portions of the tissue (see Fig. 6), which, in future iterations of our analysis pipeline, will be rejected in the patch-extraction regime using known blur detection techniques.^74^

These 2 misclassifications highlight the paramount importance of WSI quality control: even though diagnostic slides have, by definition, been determined acceptable for a medical diagnosis, digitisation artefacts present significant quality control challenges which must be overcome—either by introducing manual and or automated post-scanning quality control steps.

Another limitation of this work is that our dataset only includes instances of normal duodenal mucosa and cases determined positive for coeliac disease. While these 2 classes actually represent the vast majority of cases seen in realistic practise settings, a key step in developing our approach is to enrich our dataset with other duodenal pathologies and train the model in this more realistic setting.

The opportunity exists to extend our multiple-instance-learning approach to screen for more than one kind of duodenal pathology. Expanding our dataset to incorporate a more diverse range of diseases will allow us to investigate the feasibility of developing an approach which could screen biopsies as “normal”, “CD-positive”, or “other abnormality”. However, the rarity of other duodenal pathologies renders the prospect of building a dataset which contains enough examples for *meaningful* machine-learning research challenging.

Another interesting line of enquiry is to investigate using our approach to classify biopsies based on their modified Marsh score (as others have tried using different methodologies^64^). While a model which provides accurate Marsh grading would be valuable, such an undertaking is challenging due to the issue of poor inter-observer agreement in Marsh gradings (e.g., see Refs^46^^,^^47^) and the time intensive nature of labelling a large diverse dataset.

There is also further scope to develop our approach by reconsidering the mapping between patch- and slide-level predictions. The method we use in this work, while empirically effective, is simple and there is opportunity to explore other approaches for mapping between patch- and slide-level predictions. Investigating the benefit of such techniques will be made practical with a larger and more diverse dataset.

Given the majority of duodenal biopsies are determined to be normal, it follows that considerable time savings are possible with a model which simply screens for normal duodenal mucosa and allows pathologists to restrict their focus to abnormal cases requiring further attention.

We have demonstrated that our model can classify WSIs as normal or positive for coeliac disease with high accuracy, precision, and recall. We have also shown promising evidence that our approach generalises to new tissue samples outwith the training set, as well as samples obtained from a source completely independent from the training source. These are essential requirements for any future diagnostic algorithms (or computational methods) underpinned by this work.

The following are the supplementary data related to this article.

Supplementary material 1 Image 1

Supplementary material 2 Image 2

Supplementary material 3 Image 3

Supplementary material 4 Image 4

Supplementary figure 1 Image 5

## Authors’ contributions

J.D and B.A.S have contributed equally to the development of the WSI-processing and analysis pipeline. J.D wrote this manuscript, and B.A.S, M.J.A. and E.J.S commented on multiple revisions of it. S.C.E. collated and scanned all of the slides from Addenbrooke’s Hospital. A.S. and O.M.C. contributed to the preliminary processing of the data from Heartlands Hospital and discussions concerning the conception and planning of this project. J.L.W. compiled the metadata for the cases from Heartlands Hospital. G.L. reviewed and confirmed the diagnoses in all of the cases from Heartlands Hospital, which were cut, stained and scanned by H.B. J.D.G. and C.B.S. gave advice in early discussions concerning this work, and C.B.S. kindly facilitated access to computer resources. M.J.A. routinely provided specialist gastrointestinal pathology advice throughout this work. E.J.S. conceptualised, initiated and guided both this project and the creation of the data set.

All authors have been given the opportunity to review and approve this manuscript.

## Ethical approval

All slide scans (and accompanying fully anonymised patient data) were obtained with full ethical approval (IRAS: 162057; PI: Dr E. Soilleux).

## Declaration of interests

The authors declare the following financial interests/personal relationships which may be considered as potential competing interests:

The following authors are shareholders in Lyzeum Ltd: Elizabeth Soilleux, Mark Arends, Carola-Bibiane Schönlieb and Julian Gilbey.
