# Supplementary figures and images for: Multiple-instance-learning-based detection of coeliac disease in histological whole-slide images
Source: J Pathol Inform. 2022 Oct 28;13:100151. doi: 10.1016/j.jpi.2022.100151 (PMC9808019; doi:10.1016/j.jpi.2022.100151)

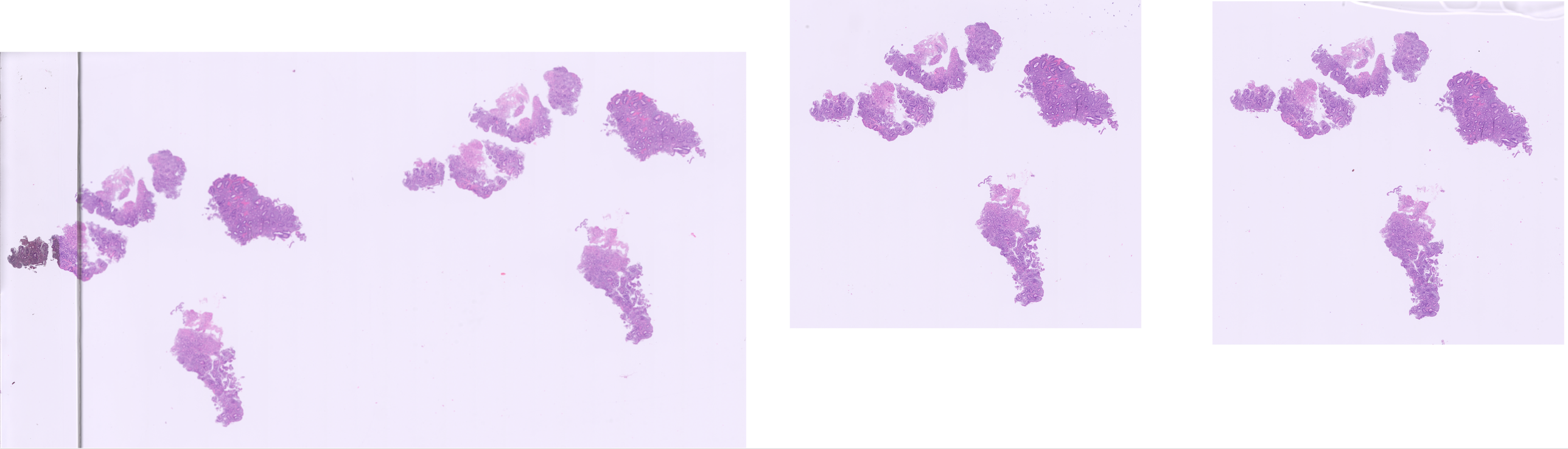

Supplement: Supplementary file 1 — Supplementary material 1 [file mmc1.zip › LYZC1110319212.png]

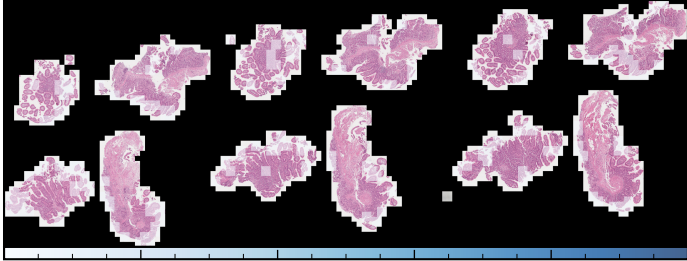

0.0

0.2

0.4

0.6

0.8

1.0

Coeliac prediction

Supplement: Supplementary file 2 — Supplementary material 2 [file mmc2.pdf]

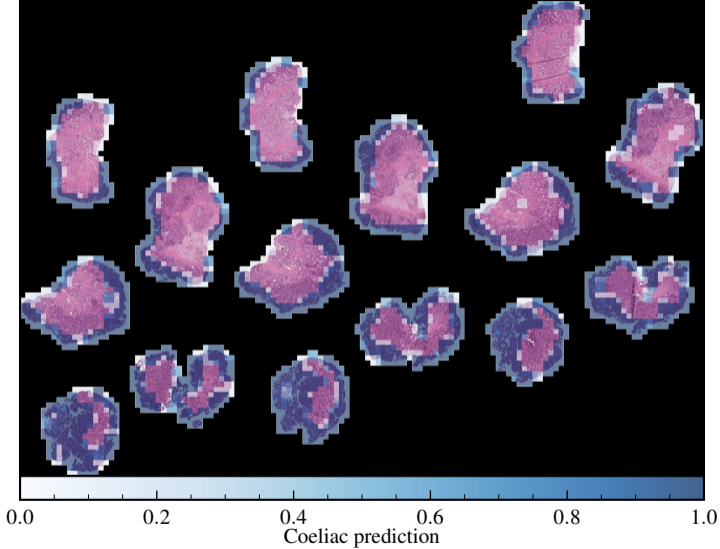

Supplement: Supplementary file 3 — Supplementary material 3 [file mmc3.pdf]

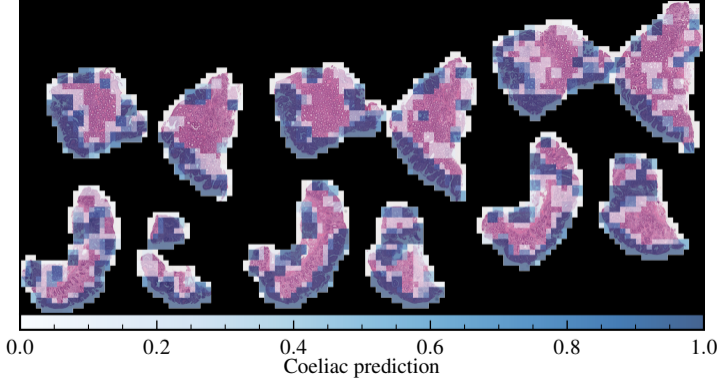

Supplement: Supplementary file 4 — Supplementary material 4 [file mmc4.pdf]

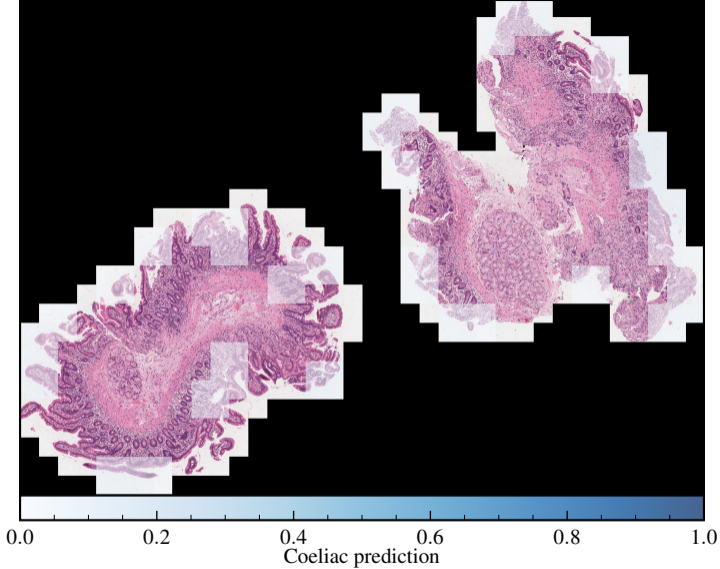

Supplement: Supplementary file 5 — Supplementary figure 1 [file mmc5.pdf]
